# Chronic Hepatitis C Virus Infection: An Ongoing Challenge in Screening and Treatment

**DOI:** 10.3390/life13101964

**Published:** 2023-09-26

**Authors:** Wei-Chu Tsai, Hseuh-Chien Chiang, Yen-Cheng Chiu, Shih-Chieh Chien, Pin-Nan Cheng, Hung-Chih Chiu

**Affiliations:** Department of Internal Medicine, National Cheng Kung University Hospital, College of Medicine, National Cheng Kung University, Tainan 704, Taiwan; cowfish5176@hotmail.com (W.-C.T.); scion456scion@gmail.com (H.-C.C.); tannoy63352@gmail.com (Y.-C.C.); slamdunk9031137@gmail.com (S.-C.C.)

**Keywords:** HCV, challenges, surveillance, strategy

## Abstract

With the advent of direct-acting antiviral agents (DAA) in the recent few years, hepatitis C virus (HCV) infection has become a curable infectious disease. Successful clearance of HCV could lead to improvement of both hepatic and extrahepatic outcomes, such as complications of cirrhosis, hepatocellular carcinoma, cardiovascular diseases, and incident diabetes. However, challenges persist in reaching the HCV elimination goals of the World Health Organization by 2030. Among these challenges are identifying those already infected or undiagnosed subjects, re-linking to the care of known but untreated HCV-infected subjects, and developing strategies to enhance treatment rates and compliance in specific or high-risk populations. In addition, issues of post-DAA viral clearance, including avoiding or preventing reinfection in high-risk populations and surveillance of hepatocellular carcinoma, are important to consolidate the treatment’s short- and long-term efficacies. In the current DAA era, treatment is the most effective prevention strategy not only in its excellent efficacy and safety but also in preventing HCV spread. All of the surveillance or measures should center on DAA treatment in clinical practice.

## 1. Introduction

**Figure 1:**
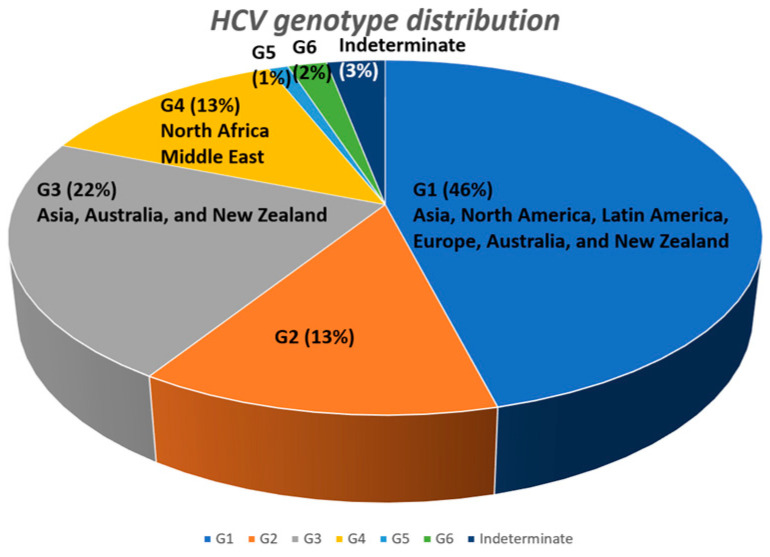
Global distribution of HCV genotypes.

Chronic hepatitis C virus (HCV) infection is a major global health problem that manifests as chronic liver diseases and relevant complications that negatively affect healthcare resources and impose a substantial economic burden. In 2020, an estimated 56.8 million individuals were chronically infected with HCV. The global prevalence of HCV infection stands at 0.7%, with regional variations that range from 2.9% in Eastern Europe to 2.6% in Central Asia [1]. There are six genotypes of HCV. On a global scale, genotype 1 accounts for 46% of HCV infections, followed by genotype 3 (22%), genotype 2 (13%), genotype 4 (13%), genotype 6 (2%), and genotype 5 (1%) [2]. In Asia, genotype 3 (39%) and genotype 1 (36%) are the most prevalent [2]. In North America, Latin America, and Europe, genotype 1 is the most dominant strain, covering 70% of cases, whereas genotype 4 accounts for 71% of the infection in North Africa and the Middle East [2]. In Australia and New Zealand, genotype 1 (53%) and genotype 3 (39%) are predominant [1,2] (Figure 1).

Since the introduction of direct-acting antivirals (DAA) in 2014, the HCV clearance rate has reached as high as over 95% [3]. However, resistance-associated substitutions can result in the emergence of resistance-associated viral variants and treatment failure [3]. Currently, two highly effective pan-genotypic DAA, especially velpatasvir/sofosbuvir (VEL/SOF) and glecaprevir/pibrentasvir (G/P), have been widely used worldwide. The effectiveness of DAA is consistent between HCV variants, regardless of the presence of resistance-associated substitutions [3]. Among the 6 HCV genotypes, genotype 3 is primarily responsible for the majority of HCV treatment failure. Hence, resistance analysis is recommended for DAA-failed patients with HCV genotype 3, and combination therapy of DAA plus ribavirin is advised [3]. The combination of sofobuvir/velpatasvir/voxilaprevir is the regimen of choice in non-responder or relapse patients.

Compared to the prevalence of HCV infection in 2015, there has been a declining trend over recent years. This is likely due to a combination of factors, including the cumulative time effects of the natural progression of HCV infection, the aging process, and advancements in antiviral therapy [1,4]. Nonetheless, a significant number of HCV-infected patients remain unaware of their status [1,5]. Globally, in 2019, only 21% and 13% of all patients with HCV infection received a necessary diagnosis and linked to treatment, respectively [6]. In 2020, the World Health Organization (WHO) estimated that approximately 1.575 million individuals (20 per 100,000 individuals) were newly infected with HCV, primarily via drug injection. Populations at higher risk of new infection include those contracting it through intravenous drug misuse (8%), sexual transmission (particularly among men who have sex with other men (MSM)), and unsafe healthcare practices [6,7]. Moreover, about 290,000 individuals (5 per 100,000 individuals) succumbed to HCV-related diseases in 2020, with liver cirrhosis and hepatocellular carcinoma (HCC) being the primary causes [7]. In 2016, the 69th World Health Assembly unanimously undertook a resolution targeting the global elimination of viral hepatitis by 2030. In line with this, the WHO outlined the following goals for HCV elimination: a 65% reduction in HCV infection-related death, an 80% reduction in new HCV infections, diagnosis in >90% of individuals with HCV infection, and treatment of >80% of diagnosed patients [8,9]. Yet, the proposed goals may not be reached by 2030, according to a study from the Polaris Observatory HCV Collaborators [1]. This study highlighted considerable gaps in diagnosis and linkage-to-care, coupled with low treatment uptake, as challenges to eradicating HCV infection. Furthermore, disparities in economic status across countries also influence treatment accessibility. Table 1 details the barriers to HCV elimination. As such, there is an urgent need to establish strategies that can effectively identify and treat HCV-infected patients.

## 2. Screening in Different Populations

### 2.1. Screening of Patients with Undiagnosed/Untreated Hepatitis C Virus Infection

Currently, testing positive patients for anti-HCV antibodies, followed by HCV RNA confirmation, is regarded as the standard diagnostic approach for HCV infection. Over 50% of patients with HCV infection are asymptomatic and unaware [10]. A comprehensive screening strategy is essential not only to detect patients who are unaware but also to reduce the transmission risk [11]. The American Association for the Study of Liver Diseases guidelines recommended universal HCV screening for all adults aged 18 and older [12]. For hospitalized patients, the incidence rate of HCV infection is approximately 0.7%–1% in countries like China, Italy, and Turkey, aligned with global prevalence rates [13,14,15]. A higher HCV-Ab prevalence in COVID centers was detected (5.1%) in Southern Italy [16]. Another study from China also revealed that male inpatients had a slightly higher anti-HCV antibody rate (0.91%) compared to female patients (0.85%) [13]. Further studies indicate a higher positive screening rate of over 3% in hospitalized patients aged over 55 years compared to non-hospitalized individuals [17,18]. A single-center study in an urban region of New Jersey found that residents aged 50–70 years yielded a higher positive screening rate of 6.3% [19]. Notably, residents living in rural areas often face challenges in accessing medical care and HCV testing [20]. Thus, strategies to broaden HCV screening beyond hospitals are needed.

The economic burden is a significant issue for HCV management. Before the advent of DAA therapy, the standard treatment for HCV was a combination therapy of interferon-α and ribavirin. Although this combination therapy yielded a sustained virological response (SVR) rate of approximately 36%–71% [21,22], the fear of side effects of interferon-based therapy and drug-related contraindications, including cirrhosis, kidney dysfunction, autoimmune disease, and severe mental and neurological disorders, deterred many HCV-infected patients from opting for this form of antiviral therapy. The cost of HCV treatment also poses an important issue. Interferon-based therapy costs between USD 2000 and USD 5000, while DAA regimens cost can reach USD 20000. However, the economic factors and public health policies differ across countries, so the best choice of HCV treatment should align with each nation’s policy. In general, DAA therapy is now the most preferred option for HCV treatment.

Identifying untreated but diagnosed HCV subjects is also a crucial step toward micro-elimination. To achieve this target, a four-step strategy has been proposed to address patients who are lost to follow-up: (1) collecting and sorting HCV-infected subjects, (2) resuming HCV infection care (re-linkage), (3) assessing their liver condition, and (4) initiating DAA therapy [23]. A study from the Netherlands examined patients’ medical records from 2001 to 2015 to identify patients with positive HCV immunoglobulin G, HCV immunoblot, and HCV RNA tests [24]. After excluding patients who had already been treated successfully, those with suspected chronic HCV infection were contacted by phone and invited to visit an outpatient clinic for complimentary medical evaluations, which comprised liver enzymes, HCV RNA, HCV genotype, and liver stiffness measurements using FibroScan. The results identified 1913 individuals with suspected HCV. Among these, 269 patients (14.1%) were successfully re-linked to care and subsequently achieved HCV clearance [24]. Another study, the ReLink-C program, sought to reconnect the caregivers and HCV-infected patients who were lost to follow-up [25]. Out of the 1591 contacted HCV-infected patients, only 10% of them were re-linked to care, and a mere 25% of those were treated with DAA [25]. Combining insights from these two studies suggested that only a limited number of HCV-infected subjects responded to phone calls, and an even smaller proportion of patients eventually received DAA treatment [24,25]. For patients who are unreachable by phone, alternative approaches such as in-person visits, outreach by local health institutes and local government, or other strategies are required. However, people who are injecting drug abusers (PWID) may lack permanent addresses, rendering home visits impractical. In these cases, drug rehabilitation centers could play a pivotal role in facilitating a connection between PWID and healthcare providers [26].

Patients who are undiagnosed and unaware of their HCV infection represent another target population. To achieve the goals of HCV elimination, this population must be identified, and appropriate screening strategies should be developed [27]. Within the community, education, screening, and easy accessibility of HCV treatment are the main steps toward HCV elimination. Martro et al. proposed a community outreach protocol in Pakistan, which included the establishment of public associations, adult schools, taxi schools, and civic centers [28]. Community-dwelling individuals were provided with comprehensive information regarding the risk of HCV infection and the need for HCV infection treatment. The screening rate of HCV through this community outreach intervention was 99.4%, which was higher than the national average of 50.7%. In addition, 83.3% of all patients with confirmed HCV infection were treated by DAA.

In rural areas, an organized effort is warranted for HCV screening. This involves the collaboration of hepatologists, nursing staff, and village leaders [29]. A successful model from Taiwan emphasized the significance of teamwork among village leaders who publicized the HCV elimination project and hepatologists and nurses at local clinics. Trained nurses are efficient in conducting telephone interviews, providing education, and performing necessary laboratory tests for all participating residents. Patients diagnosed with HCV infection were then directed or referred to local clinics for confirmatory tests and, if needed, treatment. Using this method, 64% of all residents were screened for HCV and subsequently received treatment [29]. In another HCV screening initiative in a southern Taiwan village [30], 3503 residents (74%) were screened for HCV, and 121 patients were diagnosed with HCV infection. Of these, 116 patients received DAA therapy, and ultimately, 111 patients (95.7%) achieved a sustained virological response (SVR). Such HCV screening endeavors have proven instrumental in the identification of HCV-infected patients in rural areas with limited medical accessibility and linking them to DAA therapy. Hence, fostering collaborations with organized medical teams in screening initiatives could enhance both the screening and linkage-to-care of HCV-infected patients in rural settings. Nevertheless, Taiwan, being a small island with a high population density, even in the rural area, presents a unique context. As such, the screening program in rural areas should be tailored based on each country’s demographic characteristics and public health policies.

In India, Mane et al. [31] reported that rapid diagnostic tests (RDTs) can serve as an alternative screening tool for HCV antibodies. RDTs such as Alere TrueLine, Flaviscreen, Advanced Quality, SD Bioline, and OraQuick use one drop of blood or an oral mucosa swab to test for anti-HCV antibodies. These tests offer the advantages of not requiring a medical professional, being simple to use, and having a wide application. All of the five RDTs demonstrated high sensitivity (86.3–99.4%) and a specificity of approximately 100% for detecting positive HCV antibodies. Their simplicity makes them easily accessible to all populations, thus facilitating HCV screening in countries with a large population of unscreened individuals [32]. RDTs can also be used to screen for HIV infection. Similarly, following a result of positive RTDs, patients should be linked to health care for further assessment and treatment [31].

In summary, a successful screening initiative hinges on a well-organized community screening project that involves a multi-disciplinary team, including public health workers, local governors, and medical professionals. Two key considerations in this context are the cost of the screening program and the availability of diagnostic tools for community-based HCV screening. Utilizing RDTs and subsequent DAA therapy, HCV transmission and disease complications can be prevented [33].

### 2.2. Hepatitis C Virus Screening in High-Risk Patients

PWID, sex workers, and MSM are the populations that have been reported to have a high risk of contracting HCV infection [34]. Factors such as low economic status, limited knowledge related to HCV infection, and no social interactions may contribute to the currently low screening rates in these populations, highlighting the need for screening programs tailored to the demographics [35,36]. A report by Fiore et al. from Italy indicated an HCV seroprevalence of 20.5% among 156 incarcerated female PWID in a prison [37]. The study further reported that 24 active HCV-infected subjects (75%) received DAA therapy, and 88.8% of them achieved an SVR. In Taiwan, a comprehensive anti-HCV antibody screening program was implemented in Yunlin Prison. Inmates testing positive for anti-HCV antibodies were directed to the prison’s clinic for DAA therapy. This approach showcased remarkable adherence and very high SVR rates (approximately 100%), emphasizing the crucial role of collaboration between officers and healthcare professionals and the benefit of on-site treatment [38]. For individuals with a history of drug injection, opioid agonist treatments (OATs), including methadone and buprenorphine, are often prescribed at drug rehabilitation centers [39]. Addressing the challenges of HCV diagnosis and treatment alongside OAT programs, Andrea et al. [40] conducted a “test-and-treat on-site” program to promote the elimination of HCV infection. Throughout the program duration, infectious disease specialists and nurses conducted on-site HCV/HIV antibody tests followed by HCV RNA quantification and provided DAA therapy for HCV-infected individuals. Over three years, there was a significant uptick in individuals tested for HCV antibodies and HCV RNA, and HCV prevalence reduced from 38% to 7%. In essence, OAT programs have shown promise in eliminating HCV infection. Collaborative efforts with drug rehabilitation centers or drop-in centers are pivotal in reaching out to PWID, who might have been overlooked in earlier HCV surveillance and treatment programs [41]. Hence, introducing on-site screening programs for inmates and drug abusers and ensuring they are linked to medical care emerges as a potentially effective strategy to counter HCV infection [42].

Sex workers are considered a high-risk group for HCV transmission and also face significant discrimination [43]. Addressing this challenge, a study by Lapadula et al. [44] carried out night-time street visits to spots where sex workers recruit customers. Through direct, face-to-face interviews, it was uncovered that nearly half of all sex workers faced hurdles in accessing healthcare. Out of 130 sex workers who were approached in person, 52% opted for HCV screening following the interview. These results underscore the potential of direct engagement as an effective method for promoting HCV screening among sex workers. A patient and tailored approach is essential when providing care to this population, which experiences a high level of discrimination and mistrust [44].

MSM represents another group at high risk for HCV infection. Wang et al. [45] conducted screening for MSM individuals at gay bars and sauna venues in Hong Kong. Trained fieldworkers approached attendees, providing them with health education and information about HCV screening. HCV-related knowledge was also disseminated via popular gay websites. Yet only 12% of the participants received HCV screening, and over 50% of MSM individuals remained unaware of their HCV status [45,46]. Similar to other high-risk populations, the MSM population urgently requires programs to enhance HCV diagnosis and surveillance.

## 3. Hepatitis C Virus Infection Treatment in Special Populations

Although DAA therapy is generally effective and safe, special caution is needed when treating certain populations. This includes patients with hepatitis B virus (HBV)/HCV coinfection, those with decompensated cirrhosis, and individuals undergoing liver transplantation [47].

### 3.1. Hepatitis C Virus Infection in Patients with Hepatitis B Virus Coinfection

Patients with HBV/HCV coinfection tend to exhibit more accelerated progression of liver disease than those with either HCV or HBV mono-infection [48,49]. This coinfection also enhances the higher risk of developing severe liver fibrosis and decompensated cirrhosis [49]. During DAA therapy, HBV reactivation may lead to liver failure, necessitating liver transplantation [50,51]. The exact mechanism of HBV reactivation remains unclear. Research indicates that HBV/HCV coinfected patients have an HBV reactivation rate ranging from 38% to 53% [52,53]. A randomized clinical trial from Taiwan demonstrated that entecavir could reduce HBV reactivation during DAA therapy [54]. In this trial, the reactivation rate of HBV was successfully reduced from 50% to a remarkable 0%. Therefore, prior to initiation of DAA therapy, patients with HCV infection should be screened for HBV infection [12]. For those patients with HBV/HCV coinfection, it is mandatory to monitor HBV DNA during DAA therapy. Furthermore, for patients with liver cirrhosis, concurrent anti-HBV therapy is recommended with DAA therapy and should be continued indefinitely.

### 3.2. Hepatitis C Virus Infection in Patients with Decompensated Cirrhosis

For patients with HCV-related decompensated cirrhosis, clearance of HCV by DAA therapy may halt the progression of liver disease. Given the well-known safety and efficacy of DAA, lines of evidence demonstrated the improvements in the model for end-stage liver disease (MELD) scores and quality of life for these patients [55,56]. However, a substantial proportion of patients did not regress or even worsen their liver conditions following clearance of HCV. Hence, whether to prioritize DAA therapy or to proceed directly to liver transplantation is a critical decision. DAA therapy before liver transplantation may stabilize or even improve liver function in a certain proportion of patients, leading some of them to delay or delist from liver transplantation. In a single-center retrospective study, 77 patients received DAA therapy before liver transplantation. Of them, 29 patients (37.7%) were removed from the waiting list, four (13%) died before liver transplantation, and 10 (35%) exhibited improved liver function with lower MELD scores [57]. However, many still experience the symptoms of liver cirrhosis, leading them to a status referred to as “MELD purgatory”. Ultimately, the severity of liver disease is the determining factor in deciding the sequence of DAA therapy and liver transplantation [58,59]. Using a MELD score of 20 as the cutoff, patients with MELD scores above this threshold are unlikely to improve after SVR and should prioritize liver transplantation [60].

### 3.3. Hepatitis C Virus Infection after Liver Transplantation

Liver transplantation is the definitive treatment for HCV-related liver cirrhosis. However, nearly all patients with HCV viremia experience HCV reinfection following liver transplantation before the DAA era [61]. Immunosuppressants may accelerate the progression of hepatitis [62]. If left untreated, approximately one-third of these patients with HCV viremia can develop graft cirrhosis, leading eventually to graft failure [63]. Initiation of HCV infection treatment shortly after liver transplantation can enhance graft survival rates and promote favorable long-term outcomes. With the advent of DAA therapy, the 1- and 3-year survival rates for patients with HCV undergoing liver transplantation increased from 88.7% to 92.4% and from 77.7% to 83.7%, respectively [64].

Drug–drug interactions between DAA and immunosuppressants are regarded as a minor concern for patients after liver transplantation [58]. Calcineurin inhibitors like cyclosporine and tacrolimus are commonly used as post-transplantation immunosuppressants [64]. DAA regimens containing a protease inhibitor may elevate the concentrations of cyclosporine and tacrolimus, necessitating a thorough evaluation of potential drug–drug interactions before initiating DAA therapy [65]. Sofosbuvir is a proven effective and safe drug for HCV in such patients [66]. A combination of sofosbuvir and velpatasvir is considered a preferred treatment option for post-transplantation HCV treatment [66].

## 4. Post-Sustained Virological Response Conditions

### 4.1. Hepatitis C Virus Reinfection

Reinfection is defined as the reoccurrence of viremia after the clearance of a previous infection [67]. Individuals who do not change their risk behaviors after the treatment of the initial infection remain susceptible to reinfection [68]. Among certain populations, such as drug abusers and MSM, reinfection following successful HCV infection treatment is a significant public health issue, presenting a challenge to achieving HCV elimination [69,70].

Rossi et al. [71] reported that PWID had a higher rate of HCV reinfection compared to non-drug abusers (2.04 vs. 0.31 per 100 person-years, respectively). The researchers also reported increased reinfection risks in those with a recent history of drug injection (<3 years before SVR), younger individuals (age < 45 years), those with HIV/HCV coinfection, and those with alcohol consumption. For those with a history of drug injection, OAT is an effective strategy both for reducing drug-related complications and treating opioid addiction [38]. Notably, daily use of opioid agonists has been demonstrated to reduce the likelihood of HCV reinfection [71].

Yeung et al. reported a two- to three-fold increase in reinfection rate among PWID (3.9 per 100 person-years) who achieved SVR by DAA [70]. The excellent efficacy and availability of DAA may inadvertently lead to a heightened reinfection rate. To counter this, it is essential to adopt harm reduction strategies, such as providing the use of safe syringe supplies, OAT for opioid use disorders, and broadening access to harm reduction resources [72]. The provision of syringe supplies can reduce viral spread among PWID, while OAT can lessen the frequency of injection and then further prevent HCV spread [73]. According to a meta-analysis conducted by Hajarizadeh et al., individuals on OAT and without a recent history of drug use had a 3.96 times lower risk of HCV reinfection compared with those with a recent history of drug use [74].

Several studies have indicated that the HCV reinfection rate is notably higher (ranging from 1.88 to 9.02 per 100 person-years) among the MSM population [75,76,77,78,79]. The variation of the reinfection risk might arise from methodological disparities and different risk behaviors across regions. Among younger MSM, factors like HIV/HCV coinfection and other traditional risk factors, such as drug injection and excessive alcohol consumption, contribute to reinfection [80]. Importantly, mental health counseling has been linked to a reduced risk of HCV reinfection among MSM [81]. Similar to drug abusers, MSM would benefit from post-treatment mental health and behavioral interventions to reduce the risk of reinfection.

### 4.2. Hepatocellular Carcinoma Surveillance after Sustained Virological Response to Hepatitis C Virus Infection

HCV infection increases the risk of HCC through several pathogenetic mechanisms. First, HCV infection triggers inflammation that can promote carcinogenesis. Second, chronic HCV infection also leads to liver fibrosis and cirrhosis, further enhancing the risk of HCC. Third, HCV-induced transcriptional reprogramming in the liver plays an important role in preneoplastic changes [81]. Thus, HCV eradication can halt inflammation and liver fibrosis progression and subsequently mitigate the HCC risk [82]. However, HCV clearance does not guarantee the complete elimination of HCC risk. Fibrosis reversal after HCV clearance is a slow process [83]. Risk factors such as alcohol abuse, nonalcoholic steatohepatitis, older age, and advanced liver fibrosis may remain and potentially lead to disease progression following SVR [84]. Moreover, certain HCV-induced alterations in transcriptional preneoplastic epigenetic and gene expression profiles can continue after HCV clearance [85].

The incidence of post-SVR de novo HCC occurrence ranges from 0.47 per 100 person-years in patients without cirrhosis to 2.99 per 100 person-years in those with cirrhosis [86]. To enhance early HCC detection, the European Association for the Study of the Liver recommends HCC surveillance in patients with advanced fibrosis and cirrhosis after SVR [87]. Other risk factors, such as age, alcohol consumption problems, and albumin and α-fetoprotein levels, should also be taken into account in planning an HCC surveillance program [88,89]. Various risk prediction models have been proposed [87,90,91]. For instance, Ioannou et al. [92] developed a multivariable model using 12 easily identifiable predictors, namely SVR (yes/no), sex, age, body mass index, and several others, aiming to better estimate the risk of HCC following SVR. The results showed that older patients without cirrhosis, low albumin levels, and low platelet count had an annual HCC risk of >1% before HCV clearance [92]. Fan et al. developed an “aMAP” HCC risk score model by incorporating only age, sex, albumin–bilirubin score, and platelet count. This simplified scoring method is reported to accurately predict a 5-year HCC trajectory irrespective of the patient’s ethnicity [87]. Nevertheless, additional validation and modifications are required to develop a precise screening strategy that can accurately identify individuals at risk of HCC.

Overall, the duration and frequency of surveillance significantly influence the cost and outcomes of HCC. A 6-month HCC surveillance might prevent diagnosis and excessive screening [93]. The combination of ultrasonography with serum α-fetoprotein measurement is a more sensitive screening strategy than ultrasonography alone [12,94]. Therefore, in patients with advanced fibrosis, cirrhosis, or a high HCC score, biannual ultrasonography combined with serum α-fetoprotein measurement is recommended following HCV clearance.

## 5. Conclusions

**Figure 2:**
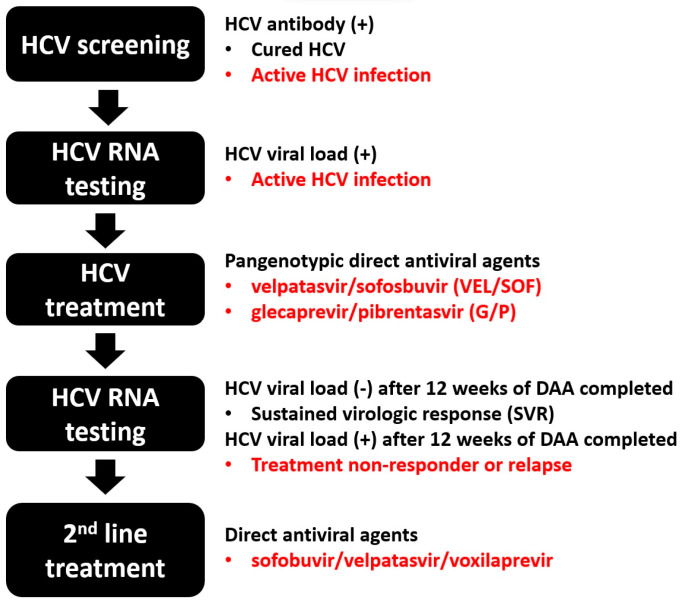
The diagnostic and treatment cascade of HCV infection.

HCV infection leads to both hepatic and extrahepatic manifestations, posing a significant social and economic burden. Across all HCV genotypes, DAA therapy can achieve a high SVR rate and exhibit excellent safety and tolerance (Figure 2). Despite these advances, barriers still exist that hinder achieving the WHO HCV elimination goals. Urgent strategies for HCV screening and healthcare linkage are imperative, especially for PWID, sex workers, MSM, and patients with HCV infection who are unaware of or untreated for their HCV infection. Collaboration between government entities and public health workers is mandatory and essential. Following HCV clearance, it is crucial to introduce education and harm-reduction behavioral interventions for high-risk populations, minimizing the risk of reinfection. Additionally, regular surveillance for HCC is necessary for patients with advanced fibrosis, cirrhosis, or other associated comorbidities.

## Figures and Tables

**Table 1:** Barriers to achieving the goals for HCV elimination of WHO before 2030.

1. Lack of effective HCV screening strategies in rural areas and in low-economic countries
2. Poor linkage to care in special populations, such as drug abusers, sex workers, and MSM
3. Need for re-linkage to care for already known but untreated HCV-infected subjects
4. Strategies needed to reduce or prevent reinfection in high-risk populations
5. Poor health knowledge of HCV infection and its management that lead to low screening rate in the general population
6. Variations of medical resources in different countries to engage in HCV elimination program
